# Synchronous Response Analysis of Features for Remote Sensing Crop Classification Based on Optical and SAR Time-Series Data

**DOI:** 10.3390/s19194227

**Published:** 2019-09-28

**Authors:** Yingwei Sun, Jiancheng Luo, Tianjun Wu, Ya’nan Zhou, Hao Liu, Lijing Gao, Wen Dong, Wei Liu, Yingpin Yang, Xiaodong Hu, Lingyu Wang, Zhongfa Zhou

**Affiliations:** 1 Institute of Remote Sensing and Digital Earth, Chinese Academy of Sciences, Beijing 100101, China; wfsunyingwei@yeah.net (Y.S.); liuhao17@radi.ac.cn (H.L.); liuweipas@163.com (W.L.); yangyp@radi.ac.cn (Y.Y.); 2 University of Chinese Academy of Sciences, Beijing 100049, China; gaolj@radi.ac.cn (L.G.); dongwen01@radi.ac.cn (W.D.); huxd@radi.ac.cn (X.H.); 3 School of Geology Engineering and Geomatics, Chang’an University, Xi’an 710064, China; wutianjun1986@163.com; 4 School of Earth Sciences and Engineering, Hohai University, Nanjing 211100, China; zhounanq@126.com; 5 College of Geography and Environmental Sciences, Guizhou Normal University, Guiyang 550001, China; wly_yu@163.com (L.W.); fa6897@163.com (Z.Z.)

**Keywords:** optical time-series data, SAR time-series data, RNN, synchronous response relationship, cloudy and rainy region, crop classification

## Abstract

Accurate crop classification is the basis of agricultural research, and remote sensing is the only effective measuring technique to classify crops over large areas. Optical remote sensing is effective in regions with good illumination; however, it usually fails to meet requirements for highly accurate crop classification in cloud-covered areas and rainy regions. Synthetic aperture radar (SAR) can achieve active data acquisition by transmitting signals; thus, it has strong resistance to cloud and rain interference. In this study, we designed an improved crop planting structure mapping framework for cloudy and rainy regions by combining optical data and SAR data, and we revealed the synchronous-response relationship of these two data types. First, we extracted geo-parcels from optical images with high spatial resolution. Second, we built a recurrent neural network (RNN)-based classifier suitable for remote sensing images on the geo-parcel scale. Third, we classified crops based on the two datasets and established the network. Fourth, we analyzed the synchronous response relationships of crops based on the results of the two classification schemes. This work is the basis for the application of remote sensing data for the fine mapping and growth monitoring of crop planting structures in cloudy and rainy areas in the future.

## 1. Introduction

Reliable agricultural information is an important basis for ensuring regional food security, and it has always been valued by countries worldwide. However, due to the scattered distributions of crops, surveys of artificial ground are too costly and difficult to adapt to meet the needs of decision-making management. Therefore, remote sensing technology can be used to obtain information on the planting areas and types of crops in a timely and rapid manner.

Since the 1970s, the United States and Europe have used remote sensing technology for a wide range of crop area monitoring and estimation systems, which not only achieved practical production guidance for agriculture but also provided an important source of information for global food trade [1]. With the development of remote sensing technology, remote sensing is becoming an important way to obtain farmland information, and it represents an important data source for crop species identification, growth analysis, and area measurement. Crop classification and planting area measurements are the main components of agricultural monitoring, and they represent two of the main topics of remote sensing research. In the optical remote sensing domain, high-resolution images can provide rich spatial and spectral information [2,3] and vegetation indices, such as normalized differential crop index (NDVI) time-series data, to improve the efficiency of crop classification [4]. Phenology characteristics are effective parameters for crop classification and can be extracted from time-series images. Based on time-series optical data, previous studies have effectively identified soybeans, rice, corn, and other major crops, and the use of this type of data has improved the efficiency of crop classification [5,6,7,8].

The traditional remote sensing techniques for crop classification, which mainly rely on optical remote sensing images, have achieved good application results. However, more than 66 percent of the Earth’s surface is regularly covered by clouds according to global cloud cover data from the International Satellite Cloud Climatology Project [9]. For example, in the cloudy and rainy regions of Southwestern China, effective optical remote images (with a cloud cover of less than 20 percent) make up less than 10 percent of annual images; thus, crop identification is difficult in such cloudy and rainy regions [10].

The all-weather and all-day imaging capability of synthetic aperture radar (SAR) ensures high repetition, high coverage, and good acquisition of specific time-phase remote sensing data. This data source is considered to be one of the most important information sources for agricultural monitoring in cloudy and rainy regions [11]. For example, many radar datasets, such as those of Radarsat [12] and Envisat-ASAR [13], are used in agriculture. Additionally, researchers have studied time-series SAR features in the classification of crops. Waske et al. [14] built a decision tree to select all available features randomly and combined them into a multiple classifier to realize crop classification in Germany based on 14 ERS-2 images. Jiao et al. [11] used 19 scenes from Radarsat-2 images to map and monitor crops in Northeastern Ontario and effectively identified five main crop types, i.e., wheat, oats, soybeans, rape, and forage. Moreover, random forests, decision trees, and other classification methods have also been widely used in crop classification [15,16,17].

However, a single-band, single-polarized radar satellite can only obtain one parameter measurement value; thus, obtaining multiple characteristic parameters of the target is difficult, which indicates the limitations of this technique [18,19]. The use of multiple bands and multiple polarization modes, as well as combinations of the two methods can effectively improve the accuracy of classification and recognition for crops. Chen et al. [20] used multiband single-polarized images, single-band multi-polarized images, and multiband multi-polarized images for crop recognition, and the results showed that the multiband multi-polarized SAR images had the best recognition ability. Jia et al. [21] used Envisat ASAR and TerraSAR-X band data for the crop identification of winter wheat and cotton, and the highest accuracy reached 91.83 percent, indicating that the use of multiband SAR data can effectively enhance the identification accuracy. Luo et al. [22] proposed a feature-level data fusion framework for hyperspectral data and SAR data. The framework effectively combines the spectral information of optical data with the structural information of SAR data to achieve the classification of features in cloud-covered regions.

Although the dielectric properties and geometrical properties of the features can be extracted from the backscattered information recorded by radar remote sensing satellites, many problems are still difficult to resolve regarding the application and full expression of information in practice. Deep learning has shown great ability in data processing [23], such as in image processing, speech recognition. Recurrent neural networks (RNNs), as a type of deep learning algorithms, are especially good at processing sequence data. Many RNNs have been used to analyze data in remote sensing fields. Dino et al. [24] built a network structure with three long short-term memory (LSTM) units and one support vector machine (SVM) layer to realize the classification of Reunion Island in a Landsat-8 dataset, and the results were highly reliable. Bahram et al. [25] used time-series RapidEye and multi-polarization Radarsat-2 data with three classic classifiers (maximum likelihood, decision tree, and random forest) in Canada for crop classification, and the results proved that it is effective to use optical and SAR data combinations for crop classification.

Moreover, deep learning has been applied to many time-series SAR datasets. Ndikumana et al. [26] demonstrated that although traditional machine learning methods, such as SVM and random forests, could be used for information extraction from Sentinel-1 time-series data, deep RNNs provided better results when processing time-series data. Wei et al. [27] superimposed Sentinel-1 time-series data onto a multichannel image and then used U-net to classify the crops in Fuyu City in a manner similar to that of the natural image classification process (convolution calculation). In addition, Zhou [28,29] extracted spatial features via the Visual Geometry Group model (VGG) to obtain feature maps and combined them as special bands with the intensity bands, and they demonstrated the effectiveness of crop classification based on an RNN structure. Their experiment was repeated, and the crop classification results are shown below.

**Figure 1:**
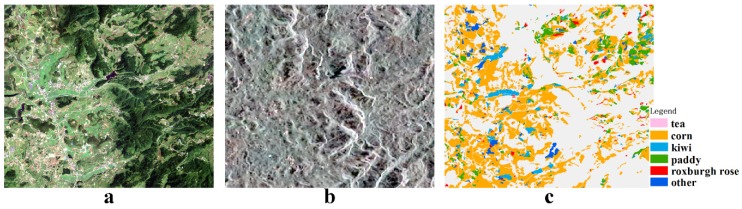
Crop classification based on synthetic aperture radar (SAR): (**a**) optical image; (**b**) SAR image; (**c**) crop planting structure.

Figure 1 shows the classification results based on Sentinel-1 time-series data achieved through the methods discussed by Zhou [28] and Sun [30] in a typical cloudy and rainy area. Based on the mentioned method, the classification accuracy of tea was only 32 percent. This proves that the accuracy of crop classification results is difficult to ensure in cloudy and rainy regions if only SAR data are used. Therefore, we performed a series of studies to improve the classification effectiveness in cloudy and rainy regions. The research system can be summarized as a crop planting study in cloudy and rainy areas under the cooperative and synchronous response mechanism of optical data and SAR data. (a) Crop planting geo-parcels were constructed based on the visual characteristics of the mosaic optical image. (b) Plot-scale crop timing features were constructed based on time-series SAR data from the study area, which enabled synergy between the optical data and SAR data. (c) Based on the response relationship between the optical data and SAR data, the time-series features of the optical data missing from cloudy and rainy areas were constructed based on time-series SAR data. The framework will be explained in the next section.

## 2. Methodology

**Figure 2:**
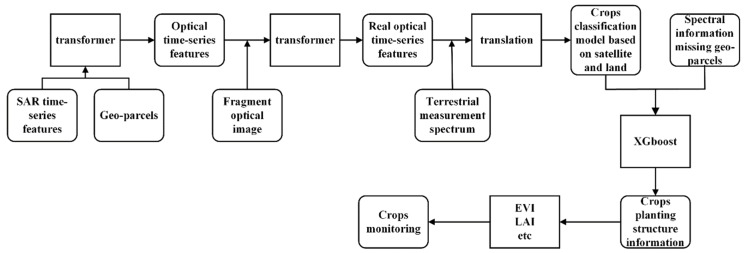
Workflow of the improved model for crop classification in cloudy and rainy regions.

Figure 2 shows our research system for crop planting structure extraction and growth index calculation in cloudy and rainy areas:

(1) Synchronous response constrction: We selected regions that have both optical time-series data and SAR time-series data to establish the time-series characteristics of the geo-parcel scale for crop planting structure mapping and then constructed the synchronous response relationship between the optical time-series data and SAR time-series data based on the transformer network.
(2) Model optimization based on fragmented optical images: The fragmented optical images in the study area were collected, and the geo-parcel features were calculated based on these fragmented images. The differences between the real optical features and the transferred optical features of the synchronous response relationship were identified based on these geo-parcels by using a rebuilt transformer network to optimize the classification model.
(3) Model optimization based on the terrestrial measurement spectrum: The terrestrial measurement spectra of the crops were collected by Analytica Spectral Devices (ASD) based on the geo-parcels. Furthermore, the differences between the terrestrial measurement spectrum and the learned optical features could be learned from the translation network, and then the real spectra could be built based on the new model.
(4) Crop class prediction for geo-parcels with missing spectral information: For the geo-parcels whose spectra were affected by mountains, mist, etc., the crop classes could be estimated by machine learning (XGBoost) [31] based on auxiliary data and geographic patterns (spatial/temporal continuity).
(5) Crop monitoring: On the basis of the crop distribution map obtained in the previous steps and the field crop site monitoring data, vegetation indices could be calculated for the purpose of remote sensing-based crop yield estimation and other applications [32,33].

**Figure 3:**
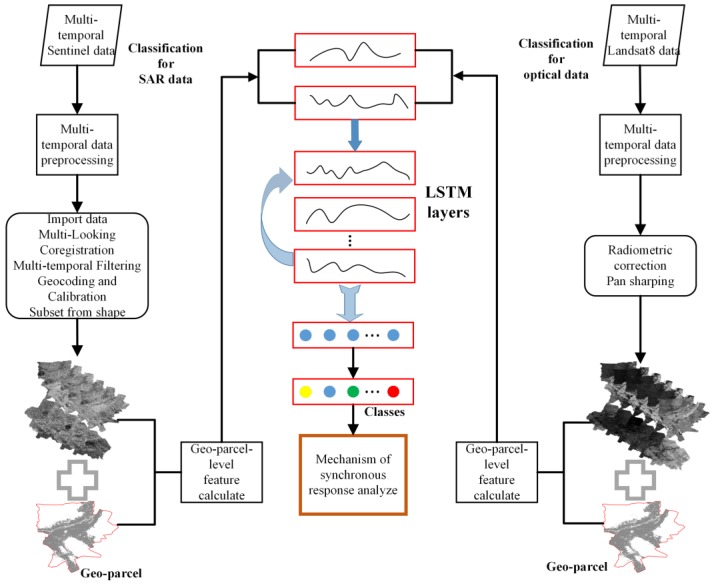
Workflow for the synchronous response relationship analysis of the crop features.

In this study, we explored the synchronous response relationships of crop features based on optical and SAR time-series data. The complete procedure can be divided into three steps: crop classification based on optical and SAR time-series data using deep learning technology, selection of the analysis samples, and synchronous response relationship analysis of the crop features. Figure 3 shows the workflow of our research. The left and right columns of the figure are based on SAR time-series data and the optical time-series data-based crop classification process, both of which are based on an RNN algorithm. Preprocessing, such as data preprocessing and feature calculation, was included in each classification process. The middle part of Figure 3 shows the relationship between the crop classifier and the synchronous response based on the characteristics of the different crop sequence characteristic curves. In the schematic diagram of this model, the blue arrow represents the stacking of the LSTM units (6 units), and the connection behind the last LSTM layer contains a dense layer and a softmax layer (blue point, second-to-last layer). The softmax layer is used to calculate and output a probability map of the crop classification (color spots, with each color representing one crop class), and the number of neurons is determined by the number of classes to be classified.

### 2.1. Geo-Parcel

The basis of this study is the geo-parcel [34,35,36], which is defined as the smallest visually perceivable spatial entity in geography. The features of geo-parcels can be represented by the spatial form and spectral information. Every geo-parcel is homogeneous and can be classified as follows: natural geo-parcels (naturally formed without traces of human activity, e.g., a permanent glacier), natural–artificial geo-parcels (naturally formed but with traces of human transformation, e.g., a barrier lake), and artificial geo-parcels (completely transformed by humans, e.g., built-up areas or farmland). The third class of geo-parcels is discussed in the next section. The geo-parcels were extracted by the method discussed in a previous study [30].

### 2.2. Recurrent Neural Networks (RNNs)

**Figure 4:**
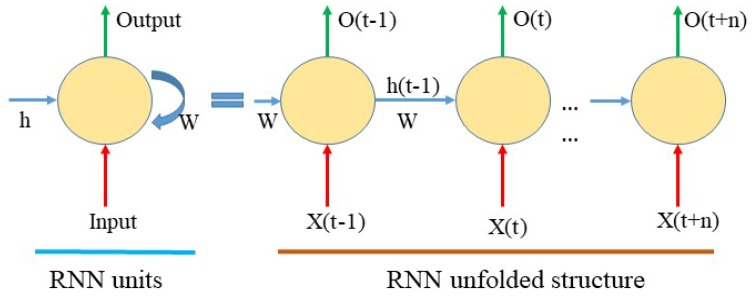
Schematic diagram of the classical recurrent neural network (RNN) structure.

RNNs are designed to learn features from sequence data, and they perform well in signal processing and speech transformation [37,38]. Compared with CNNs, RNNs mainly consider the contextual information of the sequence, which means that every state’s input covers the output of the previous state, as shown in Figure 4. In this study, an RNN structure with LSTM [39] units was used to learn the features from the established time series of optical and SAR data to classify crops.

Classical RNNs perform poorly when sequences are long due to the phenomena of vanishing and gradients. To overcome this limitation, the LSTM structure was developed. The core of a LSTM unit contains three gates—an input gate (*i_t_*), a forget gate (*f_t_*), and an output gate (*O_t_*)—which are described by Equations (1)–(6). First, the LSTM unit decides what information should be forgotten by the forget gate (*f_t_*) based on the sigmoid layer, as shown in Equation (1). Second, the retained information is selected by the input gate (*it*), as shown in Equations (2) and (3). Equation (2) shows the information (*i_t_*) retained by the sigmoid layer, and Equation (3) builds the candidate information (*A_t_*) retained by the tanh layer. Third, Equation (4) obtains a new status value (*C_t_*) based on the forget gate (*f_t_*), input gate (*i_t_*), and last status value (*C_t-1_*). Finally, (*O_t_*) impacts the new hidden state (*h_t_*), which is then exported as shown in Equations (5) and (6). The process is controlled by the sigmoid layer and the tanh layer, and the information (*h_t_*) output by this cycle is part of the information input into the next cycle.

(1) $$f_{t}=\sigma \left(W_{f}\times \left[h_{t-1},X_{t}\right]+b_{f}\right)$$

(2) $$i_{t}=\sigma \left(W_{i}\times \left[h_{t-1},X_{t}\right]+b_{i}\right)$$

(3) $$A_{t}=\tan h\left(W_{C}\times \left[h_{t-1},X_{t}\right]+b_{A}\right)$$

(4) $$C_{t}=f_{t}\times C_{t-1}+i_{t}\times A_{t}$$

(5) $$O_{t}=\sigma \left(W_{o}\times \left[h_{t-1},X_{t}\right]+b_{o}\right)$$

(6) $$h_{t}=O_{t}\times \tan h\left(A_{t}\right)$$

**Figure 5:**
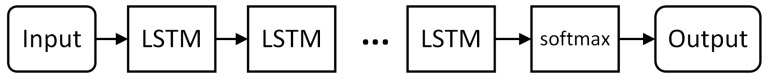
Workflow of crop classification.

Here, *σ* represents the sigmoid layer, *W* represents the different weights of every procedure, *X* represents the input in various units, *C* represents the status-describing value of the cycle, and *b* represents various bias terms. In this paper, we selected the stack type to realize the classification task. The deep architecture contains six LSTM layers to extract high-level nonlinear temporal features from time-series remote sensing datasets. Moreover, another SoftMax layer is stacked onto the last unit to perform multiclass prediction, and this layer has the same number of neurons as the class number. The structure is shown visually in Figure 5. This structure allows every hidden layer to determine the features on different time scales.

### 2.3. Discussion of the Synchronous Response Relationship

Due to different imaging methods, the characteristics of crops in optical images and SAR images are also different. However, although the same object may show different temporal characteristics in the two types of data, we can still describe the commonalities between the two temporal features in specific ways (e.g., a peak or valley value in the spectral curve). In this article, we follow this idea and try to explore the response relationship between each species in the time series according to the phenology of the crops in two datasets and generate a qualitative description.

## 3. Experiment

### 3.1. Sentinel-1 Data and Landsat-8 Data

Sentinel-1 is part of the Copernicus program of the European Space Agency (ESA) and consists of two satellites: Sentinel-1A and Sentinel-1B. These two satellites have a 6-day orbit cycle (12-day orbit individually). In this study, we used data from the interferometry wide swath (IW) mode with vertical–vertical (VV) and vertical–horizontal (VH) polarizations and with a spatial resolution of approximately 20 m. Landsat-8 data are provided by the United States Geological Survey (USGS). This satellite has 10 multispectral bands with a 30 m spatial resolution and one panchromatic band with a 15 m spatial resolution. Landsat-8 has a 16-day orbit cycle, and its data have been widely used in research on topics such as global change, agriculture, forestry, and other fields. 

### 3.2. Study Area

**Figure 6:**
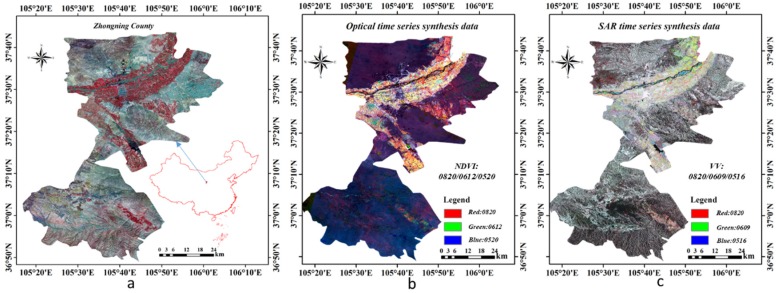
Study area: (**a**) Zhongning County; (**b**) false color image based on multitemporal NDVI data; and (**c**) false color image based on multitemporal VV polarization data.

The main content of this study is based on the classification of crops using optical data and SAR data. Therefore, the selected study area should have these two time-series datasets. Zhongning is a county that belongs to the Ningxia Hui Autonomous Region in China (Figure 6a). The average number of sunny days per year is greater than 250 days, and the conditions are suitable for both optical and SAR remote sensing images. Thus, we selected Zhongning County as the study area. In the north of this county is the Yellow River, and there are extensive mountain ranges in the southern area, which makes it unsuitable for crops. The northern part of the county belongs to the Yellow River basin and features widespread plains with fertile soil; thus, this area is the main agricultural region in Zhongning County. The crops in this area fall into two categories: food crops (e.g., corn) and cash crops (e.g., wolfberry). Therefore, this study selected the plain area in the north as the research area.

In view of the climate and crop growth cycle in the study area, we selected images from April to November 2018 as the experimental data. Additionally, we collected 17 scenes of Sentinel-1 datasets and 16 scenes of Landsat-8 images. The details of these two datasets are provided in Table 1. Furthermore, we collected validation data through a field investigation. The crop types of these geo-parcels were surveyed by the local academy of agricultural sciences.

Figure 6 also shows images of these two datasets. Figure 6b is a false color image formed by three temporal NDVI datasets (0820, 0612, and 0520) calculated from Landsat-8 data. In this image, the brighter regions are plants with more crops, and the crops are distributed along the Yellow River in Northern Zhongning County. This widespread region of crops is our research area. Figure 6c is a false image formed by three temporal backscatter intensity datasets with VV polarization from Sentinel-1 data. The brightest region here in Zhongning County is the major agricultural area, and the regions with red features are the mountainous areas, which have a strong backscatter intensity.

### 3.3. Data Preprocessing

Due to the imaging mechanism, original Sentinel-1 data should be preprocessed via data importing, multi-looking, registration, time-series filtering, geocoding, and calibration processes. In the multi-looking step, the image intensities were five range looks, the parameters of which were read from the Sentinel-1 header files. After this step, the radiation resolution of the image could be improved, and the speckle noise could be reduced. In the coregistration step, one time point was chosen as the master image, and the other images were matched to it. This process represented the preparatory work for the time-series filtering step. In the Sentinel-1 data, noise was distributed randomly which is caused by coherent interference of the system and scattering electromagnetic waves from the surfaces of objects. After transformation into an image, the noise can be efficiently removed based on the time-series filtering relationship. The purpose of geocoding is to transfer the slant distance geometry into a geographic coordinate projection based on the selected digital elevation model (DEM). Additionally, radiation correction was carried out for the Sentinel-1 images in this procedure.

### 3.4. Training Sample

Based on these two datasets and the field survey data, we built a training dataset based on geo-parcels. The format of the training data is similar to a multidimensional attribute table, and the first column is the identification number of the geo-parcels (its name could be described as “FID”). In this multidimensional attribute table, the geo-parcels’ time-series data can be understood as independent variables, and the geo-parcels’ attribute information can be thought of as dependent variables. The relationship can be simply expressed as Equation (7).

(7) $$y_{i}=\sum_{i=1}^{n}w_{i}\times x_{i}$$

Here, *y_i_* represents the crop types, *w_i_* represents the weight coefficients, which are implied coefficients that can be obtained through the established network learning. *x_i_* represents the time-series features of the geo-parcels (i.e., the geo-parcel-scale NDVI calculated from optical images or the geo-parcel-scale backscatter intensity calculated from SAR images). Sixteen features were included in the Landsat-8 training samples, and 34 features (17 VH and 17 VV) were included in the Sentinel-1 training samples. The samples were divided into six classes.

## 4. Results and Discussion

### 4.1. Experimental Results

**Figure 7:**
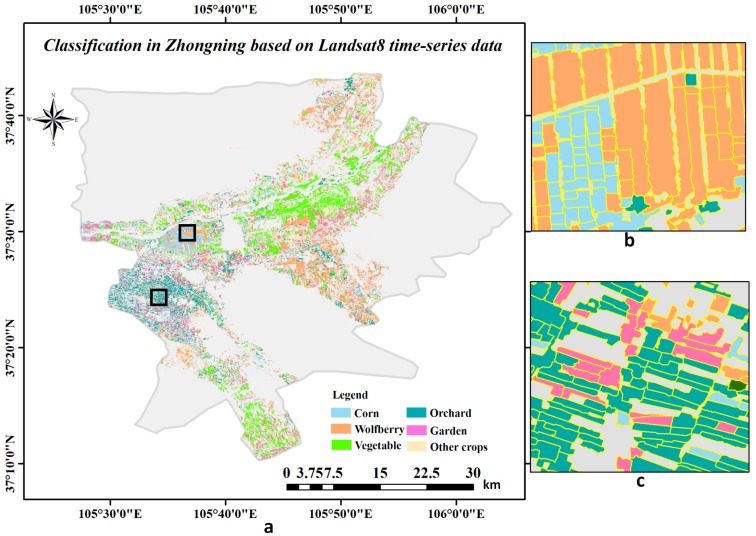

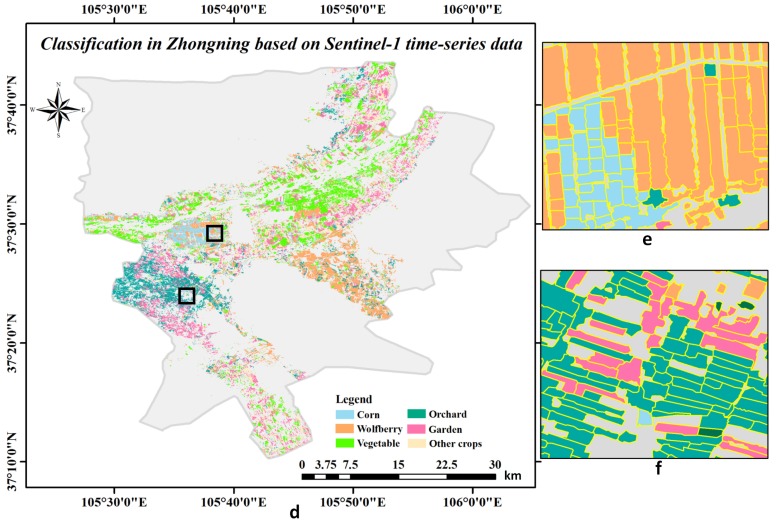
Crop classification based on Landsat-8 and Sentinel-1 datasets: (**a**) classification results based on Landsat-8 time-series data; (**b**) classification results based on Sentinel-1 time-series data; (**c**–**f**) are the enlarged figures of the black boxes in (**a**) and (**d**).

Figure 7 shows the crop mapping results of Zhongning County. More than 1600 thousandsgeo-parcels were extracted based on the richer convolutional features [40]. The figure shows that the crops can be divided into corn (we defined the geo-parcels with rotations of corn and wheat as corn geo-parcels), wolfberry, vegetable, orchard, garden, and other crops (i.e., soybeans). Additionally, the results based on the two datasets had a similar distribution. Figure 7a shows the classification results of the Landsat-8 time-series data. Figure 7b shows the results based on the Sentinel-1 time-series data. Additionally, the crops presented certain spatial distributional characteristics in the study area. First, the crops show the phenomenon of spatial aggregation, which means that crops of the same class have a high probability of being distributed in the same region. Second, the orientation of the crop distribution is in line with economic and natural laws. In detail, corn and wolfberry are mainly distributed southwest of the urban region in Zhongning County. This distribution is conducive to the effective treatment of mature crops in a timely manner. For example, first, wolfberry is distributed around densely populated regions to better ensure the effective harvesting of mature fruits. Second, vegetables are distributed in the plains on the southern bank of the Yellow River because they require more water during their growth and more agricultural facilities, such as greenhouses. Third, gardens are distributed north of the vegetables based on local policy guidance to improve the economic conditions based on the natural economic conditions. Fourth, orchards are distributed in the southwest because the geo-parcels are not suitable for growing other economic crops. The results of the classification are consistent with the crop cultivation at the time of the survey verification.

Furthermore, to quantitatively evaluate the accuracy of the classification results, we randomly selected parts of the geo-parcels from the classification results to perform a precision calculation (Table 2). The validation dataset was selected according to the approximate proportion of the number of categories in the classification result. Based on the selected validation dataset, we performed a visual interpretation to obtain the ground truth and then compared it with the classification results to calculate the accuracy of each class. The total number of the validation set was 5807, including 1571 corn areas, 1229 wolfberry areas, 1235 vegetable areas, 649 orchard areas, 706 garden areas, and 417 other areas. In terms of the classification results, the overall accuracy of the optical data was approximately 6 percent higher than that of the SAR data. Based on the test results, the Landsat-8 data were able to best identify corn with an accuracy of 94.3 percent by the LSTM, which is because corn has a double-peak structure, with the peak of the curve in summer indicating corn and the peaks in winter and spring indicating winter wheat. This double-peak structure is a core specific feature in corn-growing areas. The best classification for Sentinel-1 was wolfberry, with an accuracy of up to 91.1 percent, which was because the backscatter intensity values of wolfberry were the most aggregated. However, the other classification results of Sentinel-1 data were not as good as those of Landsat-8. The backscatter intensity values of Sentinel-1 data were more divergent than the NDVI values of Landsat-8 data. Additionally, the minority classes were difficult to map because of their lack of representation during the classifier training. Typical examples were orchards and other areas that contained forest.

### 4.2. Synchronous Response Relationship Exploration

**Figure 8:**
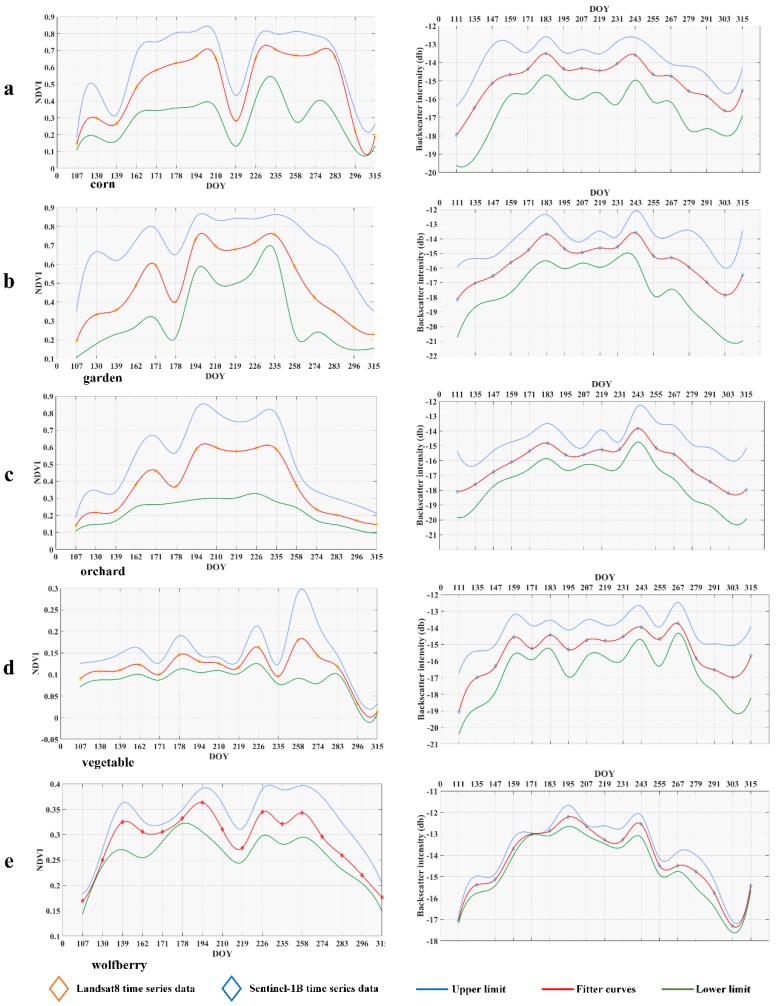
Characteristic curves of crops in Zhongning County: the same row represents the same crop. The left-column data belong to Landsat-8, and the right-column data belong to Sentinel-1 with VH polarization. (**a**) Corn and winter wheat; (**b**) garden; (**c**) orchard; (**d**) vegetable; and (**e**) wolfberry.

Based on the above classification results, we tried to explore the further synchronous response relationships. First, we performed statistical analyses according to the classification results of each category, which means that we selected the geo-parcels with consistent category labels in the classification results and further carried out sampling inspections to check the accuracy of the results in each category. In the checking procedure, we corrected misclassified geo-parcels and repeated the checking procedure until the accuracy of the randomly checked classification results in each category reached 0.9. Second, based on the selected geo-parcels, we calculated the statistical characteristics of the geo-parcels in the same category, i.e., the mean, variance, and standard deviation. Finally, we selected the mean value and the upper and lower limits as the characteristics with which to explore the synchronous response relationships of crops in Zhongning County. The fitting method of these curves was spherical regression. The curves are shown in Figure 8. Moreover, we checked the phenological information of the target crops through the statistical yearbook of Zhongning County to test our conclusions. The phenological information of these crops is shown in Table 3. The differently colored lines in Table 3 represent different crops, the start times of the lines represent the planting times of the crops and the end times represent the harvest times of the crops. For example, the first line means that corn was grown from late June to middle October and then rotated to wheat, which will be grown from late October of this year to early June of next year. A comparison showed that the phenology information obtained from the classification results based on optical data and SAR data was basically consistent with the phenology information obtained based on the statistical yearbook. Therefore, the response relationship analysis of the two datasets could be further conducted.

Based on Figure 8, we have reached the following conclusions for each crop:

Corn: The curves of the corn geo-parcels generally showed a characteristic of two peaks, which was due to the peculiar phenomenon produced by two sequentially grown crops. In March, winter wheat entered the greening period, and the NDVI continued to rise and gradually reached its peak (on the 194th day). Similarly, in late June (on the 183th day), the backscattering intensity reached the first peak, and at the 171th day, the farmland soil water content increased due to irrigation. The backscattering intensity of the SAR data produced an abnormally low value. Then, as the wheat matured and was harvested, the NDVI and backscattering intensity decreased, showing the characteristics of bare land. At approximately the 210th day, corn sowing began, and both the NDVI and backscattering intensity began to rise. At the end of September and during early October, the corn gradually matured, the chlorophyll content of the leaves decreased (at around the 290th day), and the NDVI and backscattering intensity gradually reached peaks and valleys, respectively.

Garden: Before mid-August (on the 226th day), the backscattering intensity of the SAR data was in good agreement with the NDVI data from the optical data. After entering fall (on the 226th day), the NDVI produced a jagged pattern due to pruning and irrigation. For the same reasons, the backscattering intensity exhibited the opposite jagged pattern. Then, upon entering the transplant period, the two datasets gradually showed the characteristics of bare land. The NDVI began to fall to a low point in winter, starting in mid-October.

Orchard: In the optical data, the spectral curve of the orchard showed obvious vegetation characteristics, that is, after April (on the 107th day), the reflectivity increased sharply, and the backscattering of the Sentinel-1 data showed a similar pattern. At approximately the 180th day, the NDVI from the optical data showed a low value due to summer pruning of the fruit trees and fruit thinning, but the backscatter data from Sentinel-1 did not change significantly until the end of September (on the 274th day). At the end of September (on the 274th day), the two datasets were impacted by falling leaves. The intensity was reduced, and the spectrum was characterized by the mixed characteristics of bare land and vegetation.

Vegetable: Due to their special planting structures, vegetable fields showed four peaks (on the 162th day, the 180th day, the 226th day, and the 260th day) in the optical data over the course of a year, which were generated by 4 rounds of planting. Although the curve characteristics of Sentinel-1 are not as obvious as those of Landsat-8, there is still some similarity with Landsat-8. The reason for the missing bottom of the 255th day in the Landsat-8 curves probably was that the Latdsat-8 data during this time was sparse. The vegetables in Zhongning County mainly include fast-growing vegetables, such as rapeseed and lettuce.

Wolfberry: In the optical data, wolfberry presented four peaks, with the first (on the 139th day) caused by the increased photosynthesis of leaves in spring and the remaining three (on the 194th day, the 226th day, and the 258th day) caused by the development of troughs (on the 168th day, the 215th day, and the 235th day) after three rounds of picking. For the SAR data, the VH scattering intensity of wolfberry had good aggregation, and its peak position was close to that in the optical data, i.e., on 135th day and 195th day. The different trend between 235th day and 255th day may also have been caused by the sparse Landsat-8 data.

The fitted curves show that for each crop, the reflection peaks and absorption valleys in the optical time series correspond to similar responses in the SAR time-series characteristic curves, relatively, especially in the key phenology nodes. Therefore, this synchronous response relationship based on the optical and SAR time-series characteristics of crops can be further expressed through a model in future work, which can provide important knowledge for improving the crop classification model in cloudy and rainy regions.

### 4.3. Discussion

In view of the problems involved in the fine mapping of crop planting structure in cloudy and rainy areas, we developed a research framework under the synchronous-response mechanism of optical data and SAR data. The synchronous mechanism refers to the construction of geo-parcels based on highspatial resolution optical data, followed by construction of the time features of the geo-parcel scale based on SAR data. The response mechanism refers to rebuilding the optical time-series features based on the SAR time-series features in cloudy and rainy areas. This paper conducted a preliminary study for the established research system, and we explored the synchronous response relationships for crops in Zhongning County between Landsat-8 time-series data and Sentinel-1 time-series data at the geo-parcel scale. At this scale, the same crops’ curves have shown a similar rotational characteristics simultaneously in both datasets, and this phenomenon proves that there is a response relationship, which lays the foundation for later quantitative description. 

Based on the current experiment design, we obtained the synergistic-response relationships between optical time-series data and SAR time-series data for the main crops in the study area, but it should be pointed out that there are still some problems in the research work that can be further improved in the future. First, from the data point of view, differences in the imaging time and image quality between different satellites lead to the two types of data in our study not being strictly one-to-one. For example, Landsat-8 is missing a scene (near the 267th day) relative to Sentinel-1 data, and the matching of these two data at certain times (near the 147th day, the 243th day, the 291th day and the 303th day) is lower. Moreover, there are a small number of other non-main crops in the experimental classification results of the study area (some geo-parcels are planted with alfalfa), which may result in a slight shift in the fitted crop curve that will reduce the differences between the crop curves in the study. In addition, future research also needs to mine more hidden features of images to increase the dimensions of the sample data attributes and characterize the synergistic-response relationship between the two data types on the basis of higher-precision crop planting structure mapping.

## 5. Conclusions

This paper demonstrates the research framework we developed for crop planting structure mapping in cloudy and rainy areas based on the synchronous-response mechanism of optical data and SAR data, and we have revealed the preliminary research results, but there is still much work to be done. In the future, we plan to conduct more in-depth research from the following perspectives. First, we will continue to explore the hidden features of SAR data (such as time features, grayscale features, etc.) based on the currently constructed classification model to improve the classification accuracy. Second, we will design a network that can learn the response relationships between the time-series features constructed by these two kinds of data and provide the basis for improving the crop planting structure mapping model in cloudy and rainy areas. Third, based on the time feature knowledge base established using a small amount of optical data (fragmented data) in the study area, we plan to further fit the reconstructed optical time series feature data to improve the accuracy of the reconstructed features. Fourth, in view of the problem of missing SAR signals caused by the overlap effect caused by terrain fluctuations in mountain areas, we plan to construct the crop planting structure by machine reasoning after constructing multi-dimensional attributes based on the temporal and spatial proximity of crops. Finally, based on the fine mapping of crop planting structures, we will explore the growth parameters of crops based on the characteristics of SAR time series and reconstruction and provide basic data for crop growth monitoring and yield estimation. We hope that crop planting structure mapping research under the established optical-SAR synchronous-response mechanism can overcome the problem of remote sensing technology being difficult to apply in cloudy and rainy areas due to the lack of optical remote sensing data.

## Figures and Tables

**Table 1:** Information on the experimental data.

Sentinel-1	Day of Year	Code	Landsat-8	Day of Year	Code
2018/04/22	111	1	2018/04/18	107	1
2018/05/16	135	2	2018/05/11	130	2
2018/05/28	147	3	2018/05/20	139	3
2018/06/09	159	4	2018/06/12	162	4
2018/06/21	171	5	2018/06/21	171	5
2018/07/03	183	6	2018/06/28	178	6
2018/07/15	195	7	2018/07/14	194	7
2018/07/27	207	8	2018/07/30	210	8
2018/08/08	219	9	2018/08/08	219	9
2018/08/20	231	10	2018/08/15	226	10
2018/09/01	243	11	2018/08/24	235	11
2018/09/13	255	12	2018/09/16	258	12
2018/09/25	267	13			
2018/10/07	279	14	2018/10/02	274	13
2018/10/19	291	15	2018/10/11	283	14
2018/10/31	303	16	2018/10/24	296	15
2018/11/12	315	17	2018/11/12	315	16

Sentinel-1 data can be downloaded from https://scihub.copernicus.eu; Landsat-8 data can be downloaded from https://glovis.usgs.gov/.

**Table 2:** Accuracy assessment of the crop classification results.

Class	Landsat-8	Sentinel-1
Corn (%)	**94.3**	91.1
Wolfberry (%)	89.3	**91.8**
Vegetable (%)	92.2	78.9
Orchard (%)	88.9	75
Garden (%)	82.6	77.2
**Over accuracy (%)**	**88.3**	82.1
**Kappa**	**0.86**	0.78

Best results are highlighted in bold.

**Table 3:**
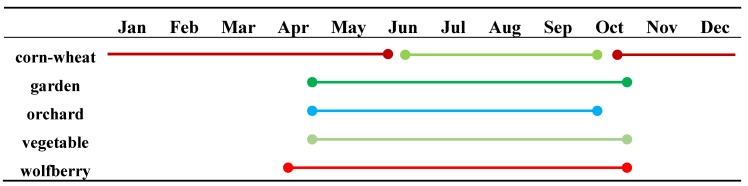
Crop phenology characteristics in Zhongning County.
